# Assessment of intrahepatic blood flow by Doppler ultrasonography: Relationship between the hepatic vein, portal vein, hepatic artery and portal pressure measured intraoperatively in patients with portal hypertension

**DOI:** 10.1186/1471-230X-11-84

**Published:** 2011-07-19

**Authors:** Li Zhang, Jikai Yin, Yunyou Duan, Yilin Yang, Lijun Yuan, Tiesheng Cao

**Affiliations:** 1 Department of Ultrasound Diagnostics, Tangdu Hospital, Fourth Military Medical University, Xi'an, China; 2 Department of General Surgery, Tangdu Hospital, Fourth Military Medical University, Xi'an, China

## Abstract

**Background:**

Abnormality of hepatic vein (HV) waveforms evaluated by Doppler ultrasonography has been widely studied in patients with chronic liver disease. We investigated the correlation between changes in HV waveforms and portal vein velocity (PVVel), the hepatic artery pulsatility index (HAPI), and also the extent of abnormal Doppler HV waveforms expressed as damping index (DI), severity of portal hypertension expressed as Child-Pugh scores and portal pressure (PP) measured directly from patients with portal hypertension (PHT) to evaluate the indicative value of abnormal HV waveforms and discuss the cause of abnormal HV waveform.

**Methods:**

Sixty patients who had been diagnosed with PHT and accepted surgical therapy of portosystemic shunts were investigated. PP was measured intraoperatively. Thirty healthy volunteers with no history of chronic liver disease were enrolled as the control group. HV waveforms were categorized as triphasic, biphasic or monophasic. DI was compared as the quantitative indicator of abnormal HV waveforms. Another two Doppler parameters, PVVel and HAPI were also measured. These Doppler features were compared with PP, Child-Pugh scores and histological changes assessed by liver biopsy.

**Results:**

In the patient group, the Doppler flow waveforms in the middle HV were triphasic in 31.6%, biphasic in 46.7%, and monophasic in 21.6% of subjects. These figures were 86.7%, 10.0%, and 3.3%, respectively, in healthy subjects. With the flattening of HV waveforms, the HAPI increased significantly (*r *= 00.438, *p *< 0.0001), whereas PVVel decreased significantly (*r *= -0.44, *p <*0.0001). Blood flow parameters, HAPI, PVVel and HV-waveform changes showed no significant correlations with Child-Pugh scores. The latter showed a significant correlation with PP (*r *= 0.589, *p *= 0.044). Changes of HV waveform and DI significantly correlated with PP (*r *= 0.579, *r *= 0.473, *p <*0.0001), and significant correlation between DI and Child-Pugh scores was observed (*r *= 0.411, *p = *0.001). PP was significantly different with respect to nodule size (*p *< 0.05), but HV-waveform changes were not significantly correlated with pathological changes.

**Conclusion:**

In patients with PHT, a monophasic HV waveform indicates higher portal pressure. Furthermore, quantitative indicator DI can reflect both higher portal pressure and more severe liver dysfunction. Flattening of HV waveforms accompanied by an increase in the HAPI and decrease in PVVel support the hypothesis that histological changes reducing HV compliance be the cause of abnormality of Doppler HV waveforms from the hemodynamic angle.

## Background

The hepatic vein (HV) is the only draining vessel in the liver, which has two supplying vessels, from the liver sinus to the inferior vena cava (IVC). The thin-walled veins are anechoic under ultrasonography, do not have valves, and can be distinguished from the portal vein. The spectrum of HVs can reflect changes in blood flow through the tricuspid valve during the cardiac cycle, leading to pulsatile changes in the spectrum during ultrasonography. The normal waveform of HVs is triphasic with two hepatofugal phases related to atrial and ventricular diastole, and a short phase of retrograde (hepatopetal) flow caused by the pressure increase in the right atrium at atrial systole. With increased stiffness in the liver parenchyma (especially around the HVs), the hepatic waveform becomes less pulsatile with no retrograde flow, and can eventually lead to a flat waveform [1-3]. The ability to detect a hepatic parenchymal abnormality by hepatic venous Doppler studies has been discussed by several authors: their opinions vary considerably. Hence, a semi-quantitative parameter DI was introduced and data showed that evaluation of the HV DI might be a useful supplemental method for assessing the therapeutic response to portal hypertensive drugs when portal pressure examination is not available [4].

The exact cause of abnormalities in HVs is controversial. One study suggested that terlipressin-induced improvement in the waveforms is evidence that a hemodynamic effect of high portal pressure rather than a fixed structural abnormality is the pathogenic mechanism responsible [5]. In the study, we evaluated the relationship between changes in intrahepatic blood flow and portal pressure (PP) measured intraoperatively in patients with portal hypertension (PHT), aiming to discuss the indicative value of HV waveform and its quantitative index DI firstly, then the cause of changes in HV waveforms both from the histological and hemodynamic angles.

## Methods

### Ethical approval of the study protocol

All subjects included in the study provided written informed consent to participate. The study protocol was approved by the ethics committee of the Fourth Military Medical University Tangdu Hospital (Xi'an, China).

### Patients and control subjects

A consecutive series of patients with previously or newly diagnosed PHT were admitted to the Fourth Military Medical University Tangdu Hospital from August 2008 to Jan 2010. Patients with hepatocellular carcinomas or intrahepatic thrombosis, or heart valvular disease or right heart disfunction which can directly affect HV Doppler pattern in the hepatic vein or IVC were excluded from the study. According to the sample size calculation of two-sample comparison, sixty patients were needed. The patients referred for surgical therapy using portosystemic shunts were finally enrolled in the study. The indications of shunt surgery for PHT patients include: patients with severe hypersplenism whose blood count should be WBC < 2.0 × 10^9^/L, PLT < 30 × 10^9^/L; patients with PHT had the history of bleeding or severe esophageal and gastric varices under the endoscopic view.

Among the PHT patients enrolled in our study, 55 patients had the history of bleeding (12 patients with more than once bleeding; 43 patients with once bleeding). Among the patients without bleeding history, 3 patients had severe hypersplenism, 2 patients had severe esophageal and gastric varices under the endoscopic view. Among 8 patients with abscent of the esophageal and gastric varices, 3 patients with severe hypersplenism were abscent of esophageal and gastric varices under the endoscopic examination, while the other 5 patients could not tolerate the whole examination, only the esophageal varices was evaluated and no varices was found in these area. The extent of esophageal and gastric varices in 12 patients was not available because of the history of rebleeding.

In order to avoid errors derived from the subjects match, 30 healthy volunteers (22 males and 8 females; age range, 20-53 years) matching with the patients from gender and age were enrolled. The subjects in control group had no history of chronic liver disease, pulmonary disease or cardiovascular disease. Laboratory tests revealed normal liver function and normal complete blood count. The severity of cirrhosis in the study group was graded according to the Child-Pugh classification [6].

### Doppler ultrasound

Doppler ultrasonographic examinations were conducted with an Acuson Sequoia 512 machine (Siemens Acuson, Mountain View, CA, USA) and a 3.5-MHz phased-array transducer. All control subjects fasted overnight before ultrasound imaging. The patients underwent ultrasound examination the day before surgery. Respiration maneuvers can alter HV flow patterns [7,8], so measurements of parameters were made during suspended respiration. Doppler HV waveforms were recorded for at least 5s with end-expiration breath holding. The middle HV was measured because it has the most consistent triphasic flow in healthy subjects and the most favorable Doppler angle. The Doppler gate was placed in the vessel 2-3 cm away from the IVC to measure the HV waveform. HV waveforms were classified as 'triphasic' (reversed flow in at least one phase), 'biphasic' (no reversed flow and with or without decreased phasic oscillation), or 'monophasic' (flat and with or without fluttering). To evaluate interobserver variation, two examiners (Yunyou Duan and Lijun Yuan) classified the recorded HV Doppler waveform tracings. The Doppler parameters we measured were consistent in all subjects.

Velocity measurements were conducted at 30-60°. The mean velocity of the portal vein (PVVel) and the hepatic artery pulsatility index (HAPI) were calculated automatically by the machine after the waveform trace for three cardiac cycles was obtained. DI (damping index) was calculated by the minimum velocity/maximum velocity of downward HV flow. To minimize variation and errors, all parameters were measured by the same observer on the same machine. They were calculated as the mean of three measurements.

Doppler examinations were undertaken by two authors (Li Zhang and Yilin Yang) without prior knowledge of the clinical and biochemical status of the study population. The reproducibility of this method was evaluated with repeated ultrasound measurements of portal venous bloodflow velocity in 10 healthy subjects over 5 consecutive days [9].

### Measurement of PP

The drugs used in general anesthesia were the same in all subjects. HR, ECG, SpO_2_, PETCO_2_, blood pressure and temperature were continuously monitored. Intraoperatively, when the blood pressure was kept constant, the right gastroepiploic vein was isolated and catheterized by a pressure gauge to measure the PP. An examiner (Jikai Yin) with 7 years of experience with PP measurement during operation performed all PP studies. The PP was estimated from three repeated measurements, and the mean value was calculated. A liver biopsy was also carried out during surgery. The biopsies were evaluated with respect to nodule size. Morphological changes were determined intraoperatively.

### Statistical analyses

Data are mean ± standard deviation. The results in patients with PHT and in healthy controls were compared using analysis of variance (ANOVA). Linear regression analysis was used to assess the correlations among all parameters. Results were considered significant at *p *< 0.05.

## Result

The mean PP measured directly from the right gastroepiploic vein was 30.02 ± 3.81 mmHg. The main clinical and pathological data for the patients at the beginning of the study are presented in Table 1.

**Table 1:** Clinical characteristics of 60 patients with PHT

Patient characteristics	
Age (median/range) (years)	47 (26-59)
Sex (F/M)	14/46
**Etiology of liver disease**	
Post-hepatitis B	44
Post-hepatitis C	12
Alcoholic	3
Cryptogenetic	3
Child-Pugh class (A/B/C)	22/30/8
Acsites (absent/light/moderate/severe)	8/42/8/2
Nodularity (small/mixed/large)	41/11/8
**Esophageal varices**	
Absent	8
Present(Small/large)	9/31
Not available	12
PP (mean ± SD) (mmHg)	30.02 ± 3.81

**Figure 1:**
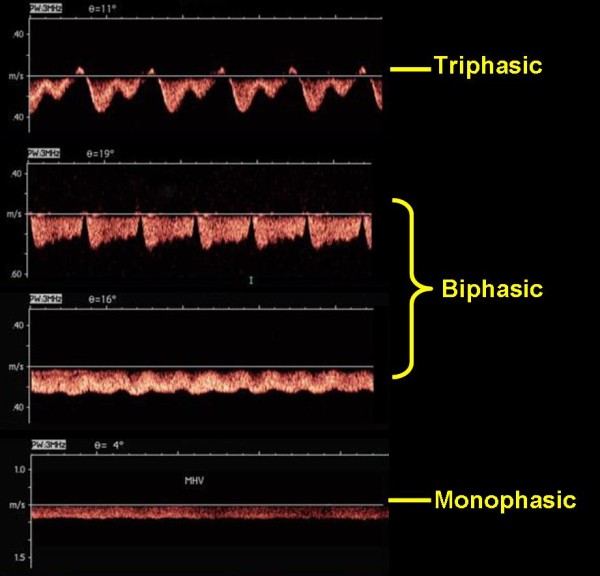
**HV waveform patterns in patients with PHT**. Triphasic pattern (two antegrade waves below the baseline and one presystolic retrograde wave above the baseline). Biphasic pattern (with absent reversed pre-systolic wave). Monophasic pattern (with a flat pattern).

The flow pattern was triphasic in 26 (86.7%), biphasic in 3 (10.0%), and monophasic in 1 (3.3%) of control subjects. Among 60 patients, the Doppler flow waveform in the middle hepatic vein was triphasic in 19 (31.6%), biphasic in 28 (46.7%), monophasic in 13 (21.6%) (Figure 1). The distribution of the HV flow pattern in the patient group according to the PP is summarized in Table 2. PP in patients with a monophasic HV waveform was much higher than in those with biphasic and triphasic waveforms (*p *= 0.004 and *p *= 0.003, respectively), but no significant difference was found between patients with biphasic and triphasic flow patterns (29.39 ± 3.40 *vs*. 28.94 ± 3.36, *p *= 0.673).

**Table 2:** Distribution of HV waveforms according to the PP in the patient group

	HV waveform		
	Triphasic	Biphasic	Monophasic
Number	19	28	13
%	31.6%	46.7%	21.6%
PP (mmHg)	28.95 ± 3.36	29.39 ± 3.40	32.92 ± 4.05
Significance of PP comparison			
	
		

**Figure 2:**
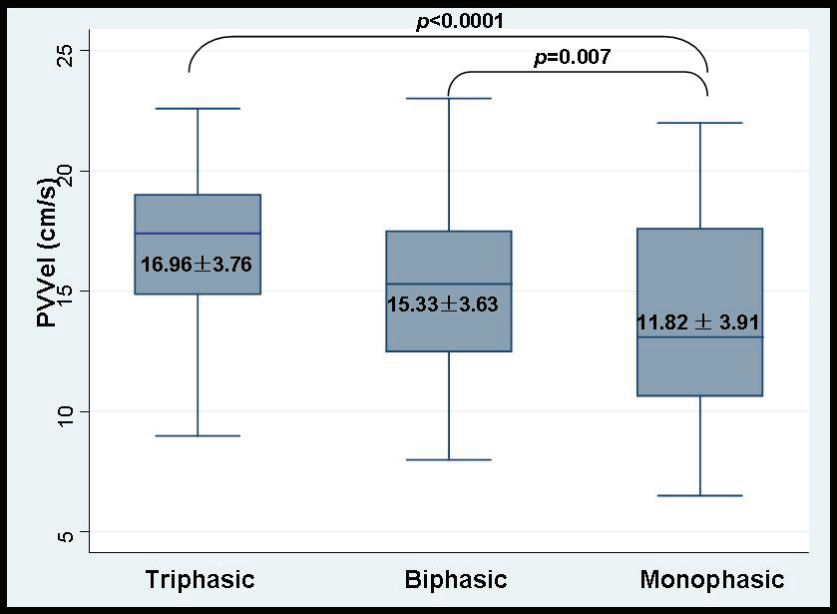
**Comparison of PVVel among different HV waveforms**.

Although PVVel was slightly lower in patients with PHT compared with healthy controls (15.08 ± 4.12 cm/s *vs*.17.14 ± 3.46 cm/s), a significant difference was not observed (*p *= 0.114), and no significant correlation was observed between PVVel and PP (*p *= 0.597). However, PVVel in patients with a monophasic pattern (11.82 ± 3.91 cm/s) decreased significantly compared with biphasic and triphasic patterns (15.33 ± 3.63 cm/s, *p *= 0.007 and 16.96 ± 3.76 cm/s, *p *< 0.0001) (Figure 2).

**Figure 3:**
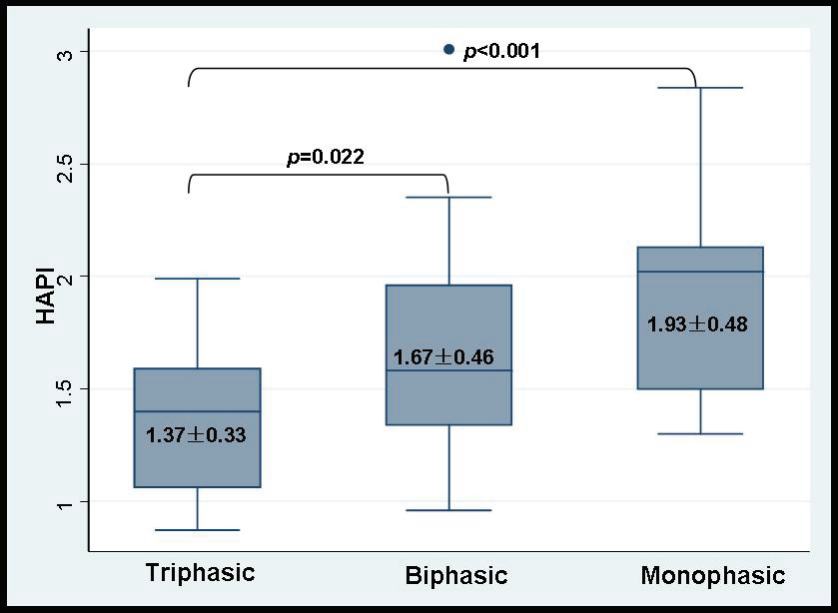
**Comparison of the HAPI among different HV waveforms**.

On average, the HAPI in the patient group was much higher than that in the healthy group (1.63 ± 0.48 *vs*. 1.17 ± 0.18, *p *< 0.0001). The HAPI in patients with monophasic and biphasic waveforms was significantly higher than that in patients with triphasic waveforms (*p *= 0.001, *p *= 0.022, respectively) (Figure 3). All the parameters about healthy subjects were summarized in Table 3.

**Table 3:** Hemodynamic results in patients and comparison with controls

Parameters	PHT group	Control group	P value
PVVel (cm/s)	15.08 ± 4.12	17.14 ± 3.46	0.114
HAP I	1.63 ± 0.48	1.17 ± 0.18	*<*0.0001*
***HV waveform***			
Triphasic	19 (31.6%)	26 (86.7%)	
Biphasic	28 (46.7%)	3 (10.0%)	
Monophasic	13 (21.6%)	1 (3.3%)	
Damping index	0.63 ± 0.15	0.44 ± 0.14	*<*0.0001*

**Figure 4:**
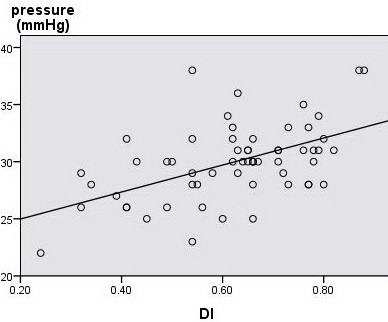
**Correlation between DI and PP in patients with PHT**.

Significant linear correlations with PP were found only in HV waveforms with *r *= 0.579 (*p <*0.0001) and with the HAPI with *r *= 0.427 (*p *= 0.001). The correlation according to the changes in HV waveforms was also observed between PVVel and the HV waveform (*r *= -0.44, *p <*0.0001), HAPI and HV waveforms (*r *= 0.438, *p *< 0.0001). Bloodflow parameters, HAPI, PVVel and HV-waveform changes showed no significant correlations with Child-Pugh scores. The Child-Pugh score showed a significant correlation with PP (*r *= 0.589, *p *= 0.044). This seemed to indicate that the weaker the liver function, the higher is the PP. However, there was no significant difference among the A, B, and C Child-Pugh scores (*p *= 0.249). HV DI was found significantly correlated with PP, namely, with higher PP, increased DI was observed*(r *= 0.473, *p <*0.0001) (Figure 4). The same correlation (with an increase of DI, the liver function weakened) was also observed between DI and Child-Pugh scores (*r *= 0.411, *p = *0.001).

In PHT patients, 68.3% of the livers had small nodules. PP was statistically different with respect to nodule size (*p *= 0.038, small/mixed; *p *= 0.009, small/large). However, changes in the HV waveform were not significantly correlated with this pathological change.

## Discussion

Various studies have been designed for evaluating the severity of liver abnormalities by Doppler ultrasound in patients with chronic liver disease [10-12]. The pattern of blood flow in the HV is one of the parameters to be evaluated. Three grades of hepatic waveforms have been described by Bolondi et al. [13] to indicate changes from the normal triphasic pattern to the flat pattern widely used in older studies of chronic liver disease [13-15]. Nowadays, more studies are carried out to determine if analyses of HV waveforms may be useful in the assessment of PHT [9,16]. In the present study, we suggest that an abnormal HV Doppler curve and quantitative index DI may be non-specific indicators of liver abnormality as well as of PP in PHT patients. Furthermore, by comparing the hemodynamic changes in the portal vein and hepatic artery and histological changes in liver parenchyma, we attempted to discuss the mechanism of abnormal HV waveform.

Even though measurement of the hepatic vein pressure gradient (HVPG) has been accepted as the 'gold standard' for assessing the degree of PHT, it is not suitable for widespread routine use because of its invasiveness [17-19]. Additionally, in the presence of increased pre-sinusoidal resistance during the cirrhotic process, the portal venous pressure can be higher than the wedged hepatic venous pressure [20]. There still was a lot of studies indicated that a good correlation between the HVPG and portal venous pressure either with a wedge catheter or a balloon catheter measured, especially in patients with alcoholic cirrhosis and hepatitis B virus (HBV) cirrhosis [21]. Considering that limitations of HVPG, and the chance we can get the directly measured portal pressure, the present study compared HV waveforms with PP measured directly in the portal venous system for the first time.

In contrast with other results [15,22], in the present study, the HV waveforms in the healthy control group also presented three types of flow patterns, whereas the triphasic HV waveform was observed in 88% of subjects. Only one person had a monophasic waveform in the HV. However, this subject had a much higher triglyceride (TG) level (8.56 mmol/L) in a subsequent blood lipid test, which may have been the cause. The two other two subjects with a biphasic HV waveform had a normal lipid profile. In patients with PHT, a monophasic HV waveform meant a higher PP, a lower PVVel, and higher hepatic artery resistance. DI in patients with PHT indicated a high likelihood of higher portal pressure and severe liver function as Child-Pugh score showed.

The exact causes of changes in Doppler HV waveforms remain unclear. Some investigators have suggested that parenchymal fibrosis and fat infiltration surrounding the wall of the HV compress the wall and reduce its compliance [23]. Other authors think that the pathogenic mechanism causing intrahepatic shunts is responsible for the abnormal waveforms [5,24]. To explore the mechanism of changes in HV waveforms, we assessed the intrahepatic changes from two aspects. Firstly, two hemodynamic parameters, PPVel and HAPI, were also assessed to evaluate the mechanism of changes in HV waveforms. The results showed that the more flattened the HV waveform, the lower the PVVel and the higher the HAPI became. Intrahepatic shunts from the hepatic artery to HVs or from the portal vein to HVs would cause increased inflow in the HVs. However, draining by HVs through vascular shunts could also decrease the vascular resistance of the supplying vessels, consequently leading to increased velocity in the portal vein and decreased resistance in the hepatic artery. However, values of PVVel and HAPI measured in the present study were not in accordance with this theory.

Secondly, with respect to pathology, we attempted to find correlations between architectural distortion in the liver parenchyma and changes in HV waveforms. Nagula et al. showed that small nodule size in the liver is also indicative of greater damage and architectural distortion of liver tissue, and further increases intrahepatic resistance. Patients with small nodules have higher PP [25]. In the present study, the relationship between PP and histological changes was consistent with the findings of Nagula et al., but direct evidence about histological changes being responsible for the changes in HV waveforms was inadequate.

In conclusion, changes in HV waveforms plus the change tendency of DI in patients with PHT can indicate the severity of PHT to a certain degree. Taking all the intrahepatic hemodynamic changes under PHT into account may provide information for investigation of the mechanism of abnormal HV waveforms. From the hemodynamic angle, we speculated that histological stiffness in the liver parenchyma around the HVs was believed to contribute to changes in HV waveforms.

The present study had several limitations. Firstly, we did not assess the effect of cardiac output and systemic vascular resistance on the hyperdynamic syndrome, which could also be a determinant of abnormalities of HV Doppler flow. Secondly, the confirmation of liver disease by liver biopsy was not carried out in healthy subjects. Hence, we could not explain exactly why there were abnormal HV waveforms in control group, we could only attribute the abnormality to abnormal blood lipid profiles. Thirdly, the histological evidence was not sufficient enough to support our hypothesis that abnormal HV waveform was caused by pathological abnormality. If the present results can be confirmed in further studies, indicative value of HV waveform and its cause can be illustrated adequately.

## Conclusions

The study highlighted the hemodynamic changes and correlations of liver blood vessels with portal pressure, Child-Pugh scores and liver morphological changes. We put more emphasis on the relationships between HV waveform changes and other parameters, aiming to assess the indicative value of HV waveform and the cause of HV abnormal changes in patients with PHT. Results showed that flattening of HV waveform was accompanied by higher PP, lower PVVel and higher HAPI. DI indicated higher portal pressure and poorer liver function. The correlations between the parameters support the hypothesis that histological changes reducing HV compliance may be the cause of abnormalities of Doppler HV waveforms in a direct and indirect way.

## Abbreviations

HV: hepatic vein; IVC: inferior vena cava; PP: portal hypertension; PPVel: portal vein velocity; HAPI: hepatic artery pulsatility index; PP: portal pressure; HVGP: hepatic venous pressure gradient.

## Competing interests

The authors declare that they have no competing interests.

## Authors' contributions

LZ undertook the ultrasound examinations and data analysis. JKY was the surgeon who measured portal pressure. YYD, YYY and LJY performed ultrasound analysis. TSC edited the article. All authors read and approved the final manuscript.

## Pre-publication history

The pre-publication history for this paper can be accessed here:

http://www.biomedcentral.com/1471-230X/11/84/prepub
